# 586 Monitoring Monocyte Anisocytosis Changes in Adult and Pediatric Burn Patients

**DOI:** 10.1093/jbcr/iraf019.215

**Published:** 2025-04-01

**Authors:** Saeed Nazemidashtarjandi, Matthew Supple, Evangeline Adjei-Danquah, Nikki Rosado, Murat Karabacak, Lael Yonker, Colleen Ryan, Robert Sheridan, Jeremy Goverman, Martin Yarmush, Daniel Irimia

**Affiliations:** Massachusetts General Hospital, Shriners Children’s; Massachusetts General Hospital; Shriners Children’s - Boston; Shriners Children’s - Boston; Massachusetts General Hospital and Harvard Medical School; Massachusetts General Hospital; Shriners Children’s - Boston and Massachusetts General Hospital; Shriners Children’s - Boston and Massachusetts General Hospital; Massachusetts General Hospital; Massachusetts General Hospital; Massachusetts General Hospital

## Abstract

**Introduction:**

Burn injuries trigger pathological immune responses. However, our ability to monitor these evolving responses is constrained by the intricate confluence of cellular, biochemical, and metabolic alterations that occur concurrently post-injury. In this study, we concentrate on monocytes, which serve as key mediators of burn-induced changes and as potential biomarkers for monitoring immune responses after injury.

**Methods:**

We enrolled adult and pediatric patients with major burn injuries (>15%) and collected blood samples twice a week during the hospitalization. We measured Monocyte Distribution Width (MDW) using a Beckman-Coulter Unicel DxH900 analyzer. As a reference, we measured the MDW changes in the presence of pro-inflammatory stimuli, e.g., lipopolysaccharide (LPS), as well as lipid mediators of inflammation resolution, e.g., resolvin D2 (RvD2). We performed single-cell RNA sequencing to identify MDW-associated transcriptional signatures in monocytes following treatment with LPS and RvD2 using HIVE CLX.

**Results:**

We enrolled N = 11 adults and N = 3 pediatric patients with major burn injuries, from whom we collected and analyzed N = 80 blood samples from adults and N = 35 from pediatric patients. In adults, we observed that MDW increased during the first week post-burn injury (MDW = 24.1), exceeding the threshold for healthy individuals of comparable age (MDW = 20.0), and gradually decreased by discharge. In non-surviving, MDW continued to rise for approximately 10 days post-injury (MDW = 28.7) and remained elevated until death. In pediatric patients with 65% TBSA burns, MDW fluctuated but remained elevated (MDW = 23.08), above the healthy threshold. However, in pediatric patients with lower TBSA burns (e.g. 45%), MDW was initially very high during the first week (MDW = 46.42), then rapidly decreased in the following week (MDW = 27.3) and continued to decline thereafter.

In blood samples from healthy donors, we found that MDW increased upon LPS stimulation (104 EU/mL, MDW= 31.8), at the higher end of values measured in patients. The addition of RvD2 (5 µM) after LPS stimulation decreased MDW significantly (MDW = 20.1, One-way ANOVA, LPS vs. RvD2 +LPS, P = 0.0002), towards values measured in healthy donors. The ex vivo addition of RvD2 to blood samples from adult burn patients with high MDW restored normal MDW values (two-tailed test, p = 0.0457, N = 4 blood samples). RNA seq analysis reveals that genes encoding proteins involved in inflammation-responsive cytokines (e.g. SLAMF7 and IL family) were significantly expressed in LPS treated monocytes and decreased in LPS+RvD2 treated monocytes.

**Conclusions:**

In patients with major burns, changes in MDW could serve as a biomarker to track inflammation and its resolution throughout the hospitalization. The ability of RvD2 to decrease MDW is novel and deserves further investigation.

**Applicability of Research to Practice:**

MDW values can be useful for monitoring hospitalized adult and pediatric burn patients.

**Funding for the Study:**

Shriners Children’s

